# Atypical viral exanthems associated with community-acquired respiratory viruses in immunocompromised pediatric patients: a case series

**DOI:** 10.3389/fmed.2025.1602533

**Published:** 2025-07-03

**Authors:** Andrea Michelerio, Alessandro Svizzero, Valeria Brazzelli

**Affiliations:** ^1^Dermatology Clinic, Fondazione IRCCS Policlinico San Matteo, Pavia, Italy; ^2^Department of Clinical-Surgical, Diagnostic and Pediatric Sciences, University of Pavia, Pavia, Italy

**Keywords:** immunocompromised pediatric patients, atypical viral exanthem, skin rash, community-acquired respiratory viruses, urgent care

## Abstract

Atypical viral exanthems can pose significant diagnostic challenges in immunocompromised pediatric patients, where rashes may mimic drug reactions, infections, or graft-versus-host disease—conditions that require different and sometimes conflicting management strategies. These fragile patients, immunocompromised because of their underlying disease or treatment, require accurate and timely diagnosis to guide appropriate care. When the etiology is infectious, recognition also has public health and infection control implications. We describe four cases of atypical exanthems in children with oncohematologic diseases or solid tumors associated with community-acquired respiratory viruses-rhinovirus or respiratory syncytial virus (RSV)-confirmed by molecular diagnostics. The rashes were transient, nonpruritic or mildly pruritic, and predominantly involved the trunk and extremities. All rashes resolved spontaneously with no change in current therapy and no invasive procedures were required. These findings underscore the role of respiratory viruses such as rhinovirus and RSV in cutaneous manifestations and highlight the utility of noninvasive molecular testing to avoid misdiagnosis and overtreatment. Reports of such viral exanthems remain scarce in the literature. Our case series expands the clinical spectrum of rashes associated with rhinovirus and RSV and underscores the importance of a multidisciplinary approach to cutaneous manifestations in pediatric oncology patients.

## Introduction

An “atypical exanthem” (AE) is a rash that differs in morphology and etiology from classic exanthems such as measles, scarlet fever, rubella, erythema infectiosum, roseola, and varicella (1, 2). The sudden onset and often extensive cutaneous involvement of AEs often prompts urgent medical evaluation. The clinical presentation of AEs is variable, ranging from mild and self-limiting to severe, life-threatening conditions that require prompt recognition and treatment. Although viral infections are the most common underlying cause, followed by drug reactions and, less frequently, bacterial or parasitic infections, determining the specific etiology remains challenging, particularly when multiple potential triggers coexist (1, 2). Pediatric oncology patients, especially those undergoing immunosuppressive therapies such as chemotherapy or hematopoietic stem cell transplantation (HSCT), are highly susceptible to atypical exanthems (3). Their immunosuppressed state complicates diagnosis, as AEs may mimic infections, drug reactions, or graft-versus-host disease (GvHD), each of which requires different management strategies (4). In this context, timely and accurate diagnosis is essential not only for optimizing patient management but also for guiding infection control and public health strategies (5). While community-acquired respiratory viruses (CARVs) such as rhinovirus and respiratory syncytial virus (RSV) are common in pediatric patients, their association with cutaneous manifestations remains poorly characterized.

We present a case series of atypical viral exanthems associated with CARVs in pediatric oncohematology patients, highlighting diagnostic challenges and outcomes. Participation in the study was voluntary, and appropriate informed consent was obtained in accordance with ethical guidelines, including those of the Declaration of Helsinki.

## Case series

At our tertiary care center, pediatric oncology patients are regularly referred to dermatology for evaluation of atypical cutaneous eruptions. We report four such cases, all characterized by self-limiting, nonpruritic or mildly pruritic erythematous-papular eruptions in the setting of impaired immune function. The rashes predominantly involved the trunk and extremities, were associated with low-grade fever, had no mucosal involvement, and resolved within a few days without deterioration in general health or significant changes in systemic therapy. In each case, molecular diagnostics identified a community-acquired respiratory virus—rhinovirus or RSV—as the likely etiologic agent, with viral clearance confirmed in follow-up testing.

All patients underwent a standardized diagnostic workup that included a thorough clinical history, physical examination, and laboratory testing. This included complete blood counts, inflammatory markers, and microbiology studies in accordance with institutional protocols for immunosuppressed pediatric patients. These included anti-streptolysin O titers and IgG/IgM serologies for pathogens such as *Mycoplasma pneumoniae*, Epstein–Barr virus (EBV), cytomegalovirus (CMV), varicella-zoster virus (VZV), HIV, parvovirus B19, and coxsackievirus. Real-time quantitative PCR was also performed on blood and/or nasopharyngeal swabs to detect viral DNA or RNA, including CMV, EBV, HHV-6 and -7, parvovirus B19, coxsackievirus, and, based on clinical suspicion, community-acquired respiratory viruses such as rhinovirus and RSV. A viral etiology was confirmed when the pathogen was identified in the acute phase, cleared in the convalescent phase, and no alternative cause was found.

### Patient 1

**Figure 1:**
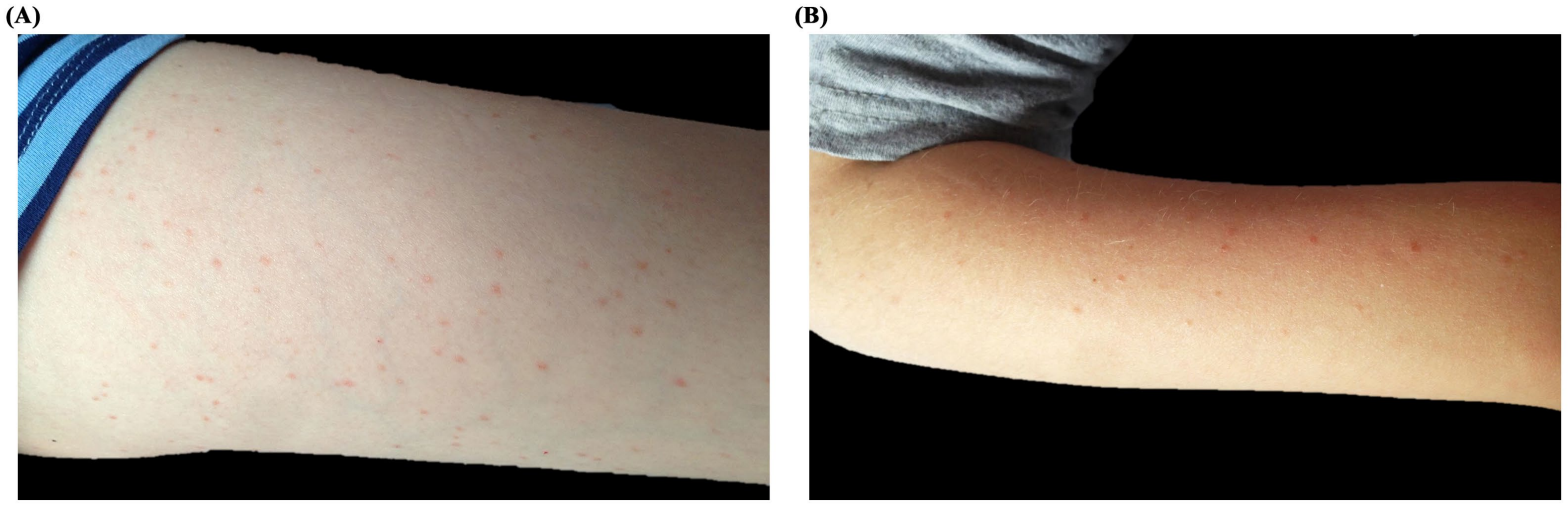
Clinical presentation of Patient 1: A 10-year-old boy with acute lymphoblastic leukemia and post-HSCT status, presenting with a papulo-erythematous rash due to rhinovirus. The rash predominantly affected the legs **(A)**, arms **(B)**, and face.

A 10-year-old boy with BCR/Abl-positive acute lymphoblastic leukemia (ALL) underwent hematopoietic stem cell transplantation (HSCT) from a partially HLA-matched donor. A few months after the transplant, he presented to the pediatric emergency department with low-grade fever (maximum temperature 38.3°C) and a widespread papulo-erythematous rash involving mainly the limbs, face, and to a lesser extent the trunk (Figures 1A,B). At that time, he was receiving prophylactic valaciclovir, cotrimoxazole, ciprofloxacin, and ondansetron, as well as intravenous immunoglobulins and granulocyte colony-stimulating factor. Differential diagnoses included viral exanthema, drug reaction, and graft-versus-host disease (GvHD). Laboratory evaluation, including blood tests, cultures, and serologic testing for varicella-zoster virus, cytomegalovirus, adenovirus, Epstein–Barr virus, parvovirus B19, and coxsackievirus, was inconclusive. To avoid destabilizing his post-transplant condition, no changes were made to his therapy. Molecular testing of a nasal swab revealed high levels of rhinovirus RNA (46,657,000 copies/mL), confirming a viral etiology. The fever resolved within 1 week and the rash resolved without intervention. A subsequent nasal swab confirmed viral clearance.

### Patient 2

A 3-year-old girl undergoing reinduction therapy for ALL developed a nonpruritic, papulo-erythematous rash involving the trunk and extremities, accompanied by low-grade fever. At that time, her treatment included cytarabine, 6-thioguanine, ciprofloxacin, and fluconazole. Laboratory tests and serologic screening were unremarkable. Real-time PCR on a nasal swab revealed a high rhinovirus RNA load (17,131,080 copies/mL). Both fever and rash resolved spontaneously within 1 week, and follow-up testing confirmed viral clearance.

**Figure 2:**
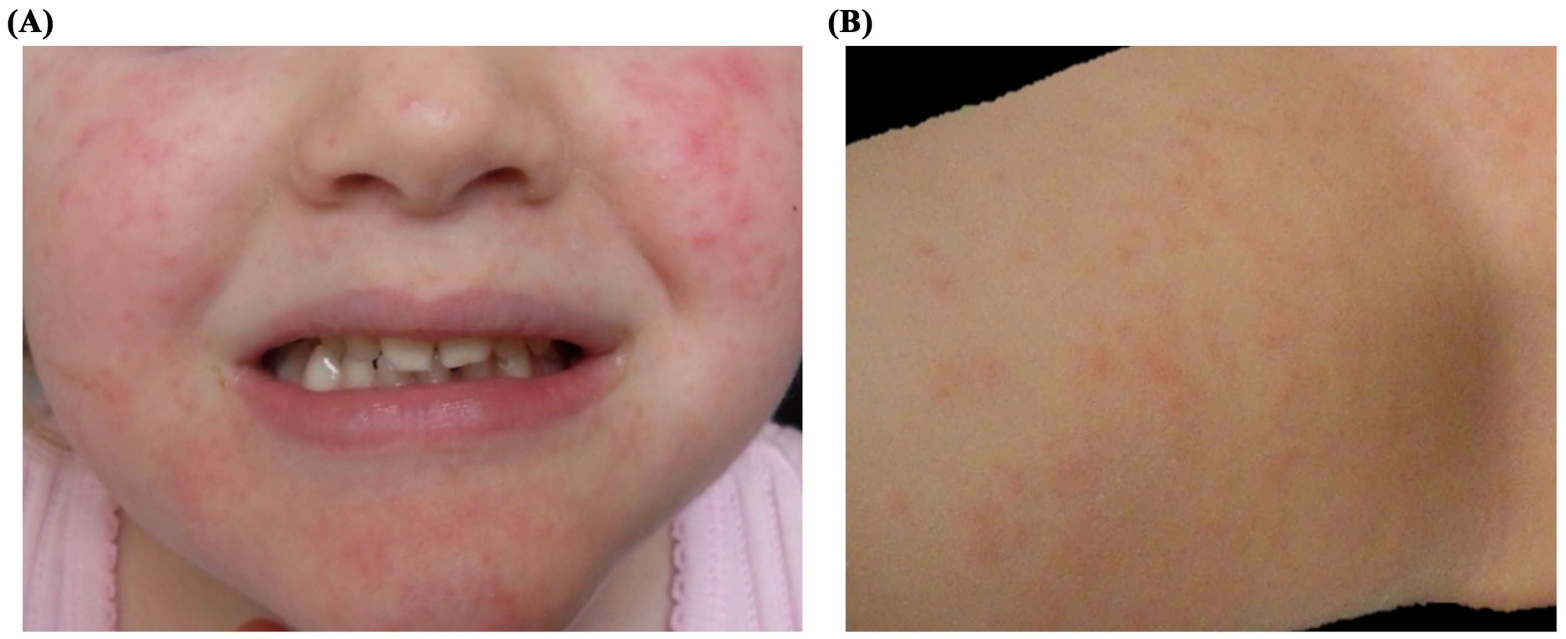
Clinical presentation of Patient 2: A 3-year-old girl undergoing reinduction therapy for acute lymphoblastic leukemia, presenting with a papulo-erythematous rash caused by rhinovirus. The rash involved the cheeks **(A)**, trunk, and limbs **(B)**.

Several months later, the patient presented with a similar rash involving the cheeks, forehead, and extremities, this time without fever (Figures 2A,B). Nasal swab testing again revealed rhinovirus RNA, but at a lower viral load (256,000 copies/mL). The rash resolved without intervention, and follow-up confirmed viral clearance.

### Patient 3

**Figure 3:**
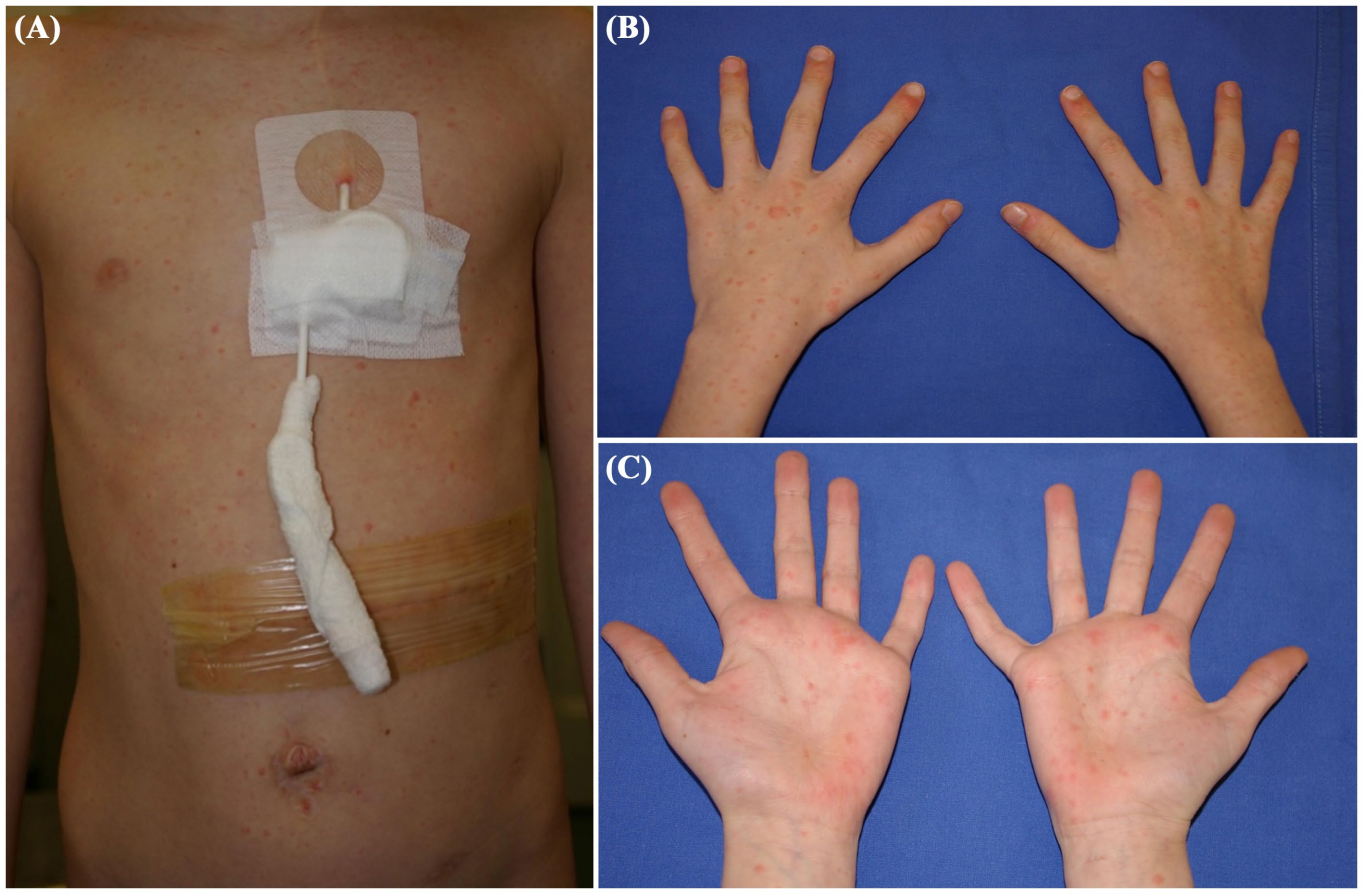
Clinical presentation of Patient 3: A 10-year-old girl with stage 3B renal cell carcinoma, showing a diffuse erythematous maculopapular rash associated with respiratory syncytial virus. The rash affected the trunk **(A)**, limbs and hands **(B,C)**.

A 10-year-old girl with stage 3B renal cell carcinoma presented with a diffuse erythematous maculopapular rash involving the trunk, extremities, hands, and feet (Figures 3A–C). The eruption was mildly pruritic and not associated with mucosal involvement. Her oncologic history included prior treatment with interleukin-2 and multiple cycles of sorafenib for abdominal recurrence. The rash presented with low-grade fever. Initial laboratory tests were unremarkable except for findings related to her underlying disease. Given the known association between sorafenib and cutaneous adverse events, including hand-foot skin reaction (6), the drug was temporarily discontinued as a precaution. Molecular testing of a nasal swab revealed RSV RNA (7,308,980 copies/mL), suggesting a viral etiology. The rash and fever resolved spontaneously within 2 weeks. Sorafenib was subsequently reintroduced without recurrence of symptoms, supporting RSV as the likely cause of the rash, and no long-term modification of therapy was required.

### Patient 4

**Figure 4:**
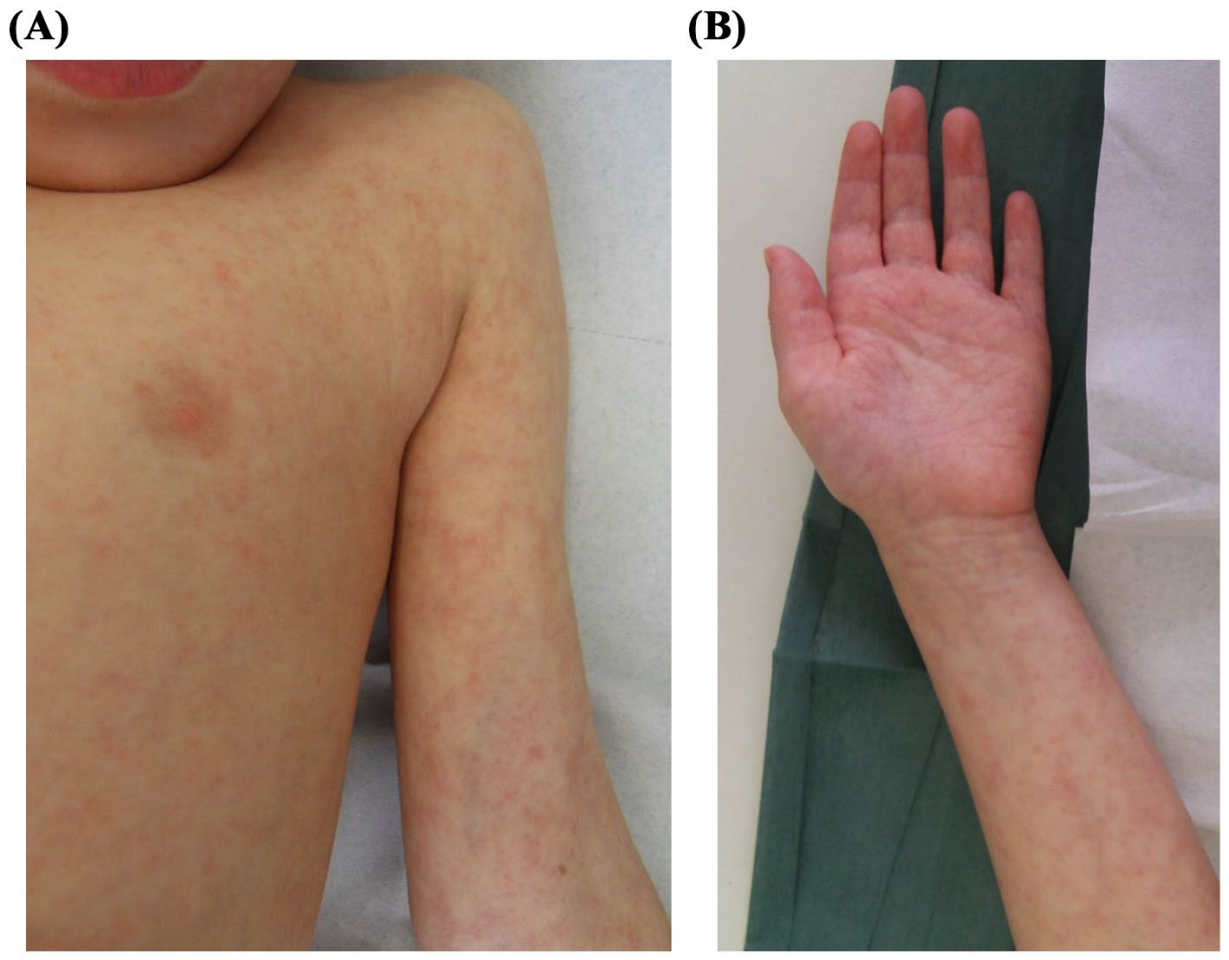
Clinical presentation of Patient 4: A 3-year-old girl with a history of aplastic anemia post-HSCT, presenting with a papulo-erythematous rash caused by respiratory syncytial virus. The rash involved the trunk **(A)**, extremities and palms **(B)**.

A 3-year-old girl with a history of aplastic anemia underwent unrelated donor hematopoietic stem cell transplantation after conditioning with fludarabine, cyclophosphamide, total body irradiation, and antithymocyte globulin. Nine months later, she presented with a nonpruritic papulo-erythematous rash involving the trunk, extremities, and palms and soles (Figures 4A,B), accompanied by low-grade fever and without mucosal involvement. At the time, she was receiving cyclosporine, trimethoprim-sulfamethoxazole, ciprofloxacin, and fluconazole, any of which could potentially cause a drug eruption. Laboratory investigations, including a complete blood count, biochemistry, blood cultures, serology, and molecular testing for respiratory viruses, were performed. Nasopharyngeal swabs tested positive for RSV RNA (3,198,630 copies/mL), suggesting an AE caused by RSV infection. The rash and fever resolved spontaneously within 2 weeks, and follow-up testing confirmed viral clearance. No changes were made to her immunosuppressive or prophylactic regimens, and her clinical condition remained stable throughout.

## Discussion

AE are common in pediatric patients and often have a viral etiology (1, 2). However, in immunocompromised children, such as those undergoing chemotherapy or hematopoietic stem cell transplantation (HSCT), these rashes pose significant diagnostic challenges due to their broad differential diagnosis, including drug reactions, graft-versus-host disease (GvHD), or manifestations of the underlying disease.

Advances in therapies such as chemotherapy, radiation and BMT have improved outcomes but also increased the risk of complications, particularly infections, in this population (7, 8). Immunosuppression, whether disease-related or therapy-induced, profoundly impairs host defense mechanisms, increasing susceptibility to infections and complicating their diagnosis (4, 9).

Viral infections, which are usually mild and self-limiting in healthy children, can cause substantial morbidity and mortality in immunocompromised patients (8, 9). CARVs, including rhinovirus and RSV, are common in this group (10, 11) and unlike other pathogens such as HSV, VZV and CMV, no effective prophylaxis currently exists, leaving these viruses as a persistent threat (11).

While CARVs typically cause respiratory disease, their potential to present with isolated dermatologic manifestations remains underrecognized. Rhinovirus, traditionally associated with upper respiratory tract infections in healthy individuals, emerged as the causative agent in two cases (12–14). Despite its established role in respiratory infections, rhinovirus-associated cutaneous manifestations remain sparsely reported (15, 16). The two cases herein provide further evidence of its potential to induce rashes in immunocompromised patients, even in the absence of respiratory symptoms.

RSV is a leading cause of acute lower respiratory tract infections worldwide, particularly in infants and young children, frequently resulting in hospitalization due to bronchiolitis and pneumonia (17, 18). Although predominantly linked to respiratory disease, extrapulmonary manifestations of RSV, including cardiovascular complications, seizures, hepatitis, and scarlatiniform eruptions involving the trunk and face, have been documented (19–21). In our series, RSV infection was associated with maculopapular rashes without significant systemic involvement in two cases. Notably, immunocompromised pediatric cancer patients may require interventions ranging from symptomatic management to intensive therapies, including oxygen supplementation and mechanical ventilation in severe respiratory cases (22).

In immunocompetent children, viral exanthems are typically diagnosed clinically in primary care settings, with laboratory confirmation rarely sought due to the self-limited nature of the disease and the low risk of complications. However, in immunocompromised hosts, even benign-appearing rashes may raise concern for serious disease, necessitating a more thorough diagnostic approach. Molecular diagnostics, particularly PCR testing of nasopharyngeal swabs, played a critical role in confirming viral etiology and guiding management decisions in our cases. Identification of viral RNA during acute illness and subsequent clearance at follow-up strengthened causal attribution and minimized unnecessary diagnostic procedures or therapeutic interventions. In particular, biopsies, while potentially informative, should be carefully considered in this patient population due to the inherent risks of infection and delayed wound healing associated with immunosuppression. However, clinicians should remain aware of the limitations of PCR, including differentiation of primary infections from reactivations.

Clinically, some features of these viral exanthems may help distinguish them from other causes (23–25). Recognition of a recurrent clinical pattern, such as a self-limiting, nonpruritic or mildly pruritic, erythematous-papular rash associated with low-grade fever involving the trunk and extremities and sparing the mucous membranes, may raise clinical suspicion of a viral etiology. Increased awareness of CARV-associated exanthems may allow clinicians to take a conservative, targeted approach, especially when close observation is possible. Dermatologic consultation can contribute significantly not only to accurate diagnosis, but also to resource rationalization and avoidance of unnecessary interventions-a strategy appreciated by patients’ families, who reported reassurance regarding the benign course of exanthems and the stability of ongoing treatments. In conclusion, the multifactorial nature of atypical exanthems in pediatric oncology underscores the importance of a comprehensive, multidisciplinary approach. Collaboration between dermatologists, infectious disease specialists, and oncologists ensures accurate diagnoses, minimizes unnecessary treatment adjustments, and avoids inappropriate therapies. Despite the inherent limitations of a small, retrospective case series, our observations highlight CARVs as potential dermatologic pathogens in immunocompromised pediatric populations. Increased awareness and timely molecular diagnosis may prevent unnecessary biopsies, empiric treatments, and diagnostic delays, thereby optimizing patient outcomes. CARVs should be actively considered in the differential diagnosis of benign exanthems in immunocompromised children, and awareness of their dermatologic presentation may improve patient care.

## Data Availability

The datasets for this article are not publicly available due to concerns regarding participant/patient anonymity. Requests to access the datasets should be directed to the corresponding author.
